# Global Research Trends of Ferroptosis: A Rapidly Evolving Field With Enormous Potential

**DOI:** 10.3389/fcell.2021.646311

**Published:** 2021-04-29

**Authors:** Haiyang Wu, Yulin Wang, Linjian Tong, Hua Yan, Zhiming Sun

**Affiliations:** ^1^Clinical College of Neurology, Neurosurgery and Neurorehabilitation, Tianjin Medical University, Tianjin, China; ^2^Tianjin Key Laboratory of Cerebral Vascular and Neurodegenerative Diseases, Tianjin Neurosurgical Institute, Tianjin Huanhu Hospital, Tianjin, China; ^3^Department of Orthopaedic Surgery, Tianjin Huanhu Hospital, Tianjin, China

**Keywords:** ferroptosis, bibliometric analysis, WoSCC, VOS viewer, CiteSpace

## Abstract

**Background:** Ferroptosis is a newly proposed form of programmed cell death, and accumulating evidence suggests that it plays an essential role in the development of multiple diseases, especially cancers and neurodegenerative diseases. Since officially named in 2012, research on ferroptosis has grown rapidly. There are previous reviews focused on the research progress of ferroptosis from a certain aspect, but no bibliometric studies summarizing this field as a whole. This study aimed to assess the scientific output and activity regarding ferroptosis research from a global perspective.

**Methods:** Publications related to ferroptosis from 2012 to 2020 were identified and selected from the Web of Science Core Collection. Excel 2019 and GraphPad Prism 8.0 was used to analyze quantitative variables including number of publications and citations, H-index, and journal citation reports. VOS viewer and CiteSpace were used to perform co-authorship, co-citation, and co-occurrence analysis of countries/institutes/authors/keywords.

**Results:** A total of 1,285 publications on ferroptosis research were identified. The literature on ferroptosis had been continuously growing since 2012, and the expansion might continue at a rapid pace in the following years. China contributed the greatest proportion (43.74%) of ferroptosis publications, and the United States ranked first in the number of citation frequency (20,980 times) and H-index (70). B. R. Stockwell, D. L. Tang, and R. Kang were key researchers. The journal *Cell Death Disease* published the highest number of articles, with 42 articles. All the keywords could be divided into two clusters: cluster 1 (pathway and mechanism) and cluster 2 (treatment and effect). In terms of potential hotspots, keywords with the strong bursts and still ongoing recently were “neurodegeneration” (2017–2020), “chemotherapy” (2017–2020), “NF-kappa B” (2017–2020), and “photodynamic therapy” (2018–2020).

**Conclusion:** There will be a dramatically increasing number of publications on ferroptosis research based on the current global trends. China has made significant progress in ferroptosis research, but the United States is actually dominated in this field. More focus will be placed on neurodegeneration, chemotherapy, nuclear factor κB, and photodynamic therapy, which may be the next popular topics in ferroptosis research.

## Introduction

Ferroptosis is an iron-dependent form of programmed cell death, primarily characterized by impaired cystine uptake into cells, subsequent glutathione depletion, iron-dependent lipid peroxidation, and the release of damage-associated molecular patterns (Dixon et al., 2012; Stockwell et al., 2017). In 2012, the term *ferroptosis* was first coined by Dixon et al. (2012), as the discovery of RAS-selective lethal small molecule erastin could selectively induce a unique nonapoptotic form of cell death in cancer cells, which could also be inhibited by iron chelators or lipophilic antioxidants such as ferrostatin-1. According to the recommendations of the Nomenclature Committee on Cell Death in 2018, ferroptosis was defined as one kind of the regulated cell death mainly caused by glutathione peroxidase 4 (Gpx4) disorder in regulating intracellular oxidative imbalance (Galluzzi et al., 2018). Emerging evidence indicates that multiple molecules and signaling pathways associated with oxidative stress have been linked to regulation of ferroptotic progress (Wang et al., 2020). Accumulation of free iron and that of toxic lipid peroxides are the two major hallmarks of ferroptosis, which is different from other types of cell death (Chen X. et al., 2020; Chen et al., 2021).

Over the past few years, the cumulative evidence indicated that impaired ferroptosis is implicated in the development of various pathological conditions, most notably cancer (Zielke et al., 2018; Yang et al., 2019, 2020), neurodegenerative diseases (Reichert et al., 2020; Sun et al., 2020), tissue ischemia/reperfusion injury (Tuo et al., 2017; Li et al., 2019), craniocerebral trauma (Tang et al., 2020), and inflammation (Proneth and Conrad, 2019). Therefore, it is a promising field to develop potent ferroptosis inducers or inhibitors with high potential for preventing and treating the aforementioned diseases, as well as several other underlying disorders related to ferroptosis. Taking oncologic diseases as examples, several scholars have proposed potential strategies for cancer treatment, and several agents have shown the ability to trigger ferroptosis of cancer cells by acting on system xc (–), Gpx4, and endoplasmic reticulum (ER) homeostasis (Bersuker et al., 2019; Liang et al., 2019; Lelièvre et al., 2020).

In recent years, ferroptosis has gained considerable attention in various research areas, and many academic journals have published articles on ferroptosis research. However, few attempts have been undertaken to investigate the scientific output and current status systematically in this field from a global perspective. As a consequence, it is necessary and essential to adopt a suitable visualization method to reveal the global status, future research trends, and hotspots in ferroptosis research.

Bibliometrics analysis is a good choice to analyze the knowledge structure of a scientific domain and development trends in research activity over time (Synnestvedt et al., 2005; van-Eck and Waltman, 2010). It is a feasible method to evaluate the publications on a specific subject qualitatively and quantitatively and has been widely used in various fields to estimate productivity and international collaboration of countries, institutions, and the authors (Gao et al., 2019; Deng et al., 2020; Yao et al., 2020). Yet, the application of bibliometric analysis is still in the early phase in the fields of cell and molecular biology. And not many studies using this method have been published outside of the ion channels (Zhao D. et al., 2018), mesenchymal stem cells (Zhao J. et al., 2018), long noncoding RNAs (Miao et al., 2017), and sigma-1 receptor fields (Romero and Portillo-Salido, 2019). To the best of our knowledge, a specific bibliometric analysis of ferroptosis research has not yet been performed.

In the present study, we performed a bibliometric analysis to systematically evaluate the ferroptosis studies from 2012 to 2020. We take advantage of this new technique, which combines mathematical and statistical methods with data visualization to estimate the publication pattern of ferroptosis research worldwide; to assess the cooperation pattern between countries, institutions, and the authors; and to identify research trends and hotspots in this field.

## Materials and Methods

### Source Database

In this study, we chose the Science Citation Index (SCI) Expanded of Web of Science Core Collection (WoSCC) database as the data source. The WoSCC database is regarded as one of the most comprehensive, systematic, and authoritative databases, which contains more than 12,000 influential high-quality journals throughout the world and has been widely used for scientometric analysis and visualization of scientific literature in a substantial number of studies (Miao et al., 2017; Romero and Portillo-Salido, 2019; Zhang et al., 2020).

### Retrieval Strategies

**FIGURE 1:**
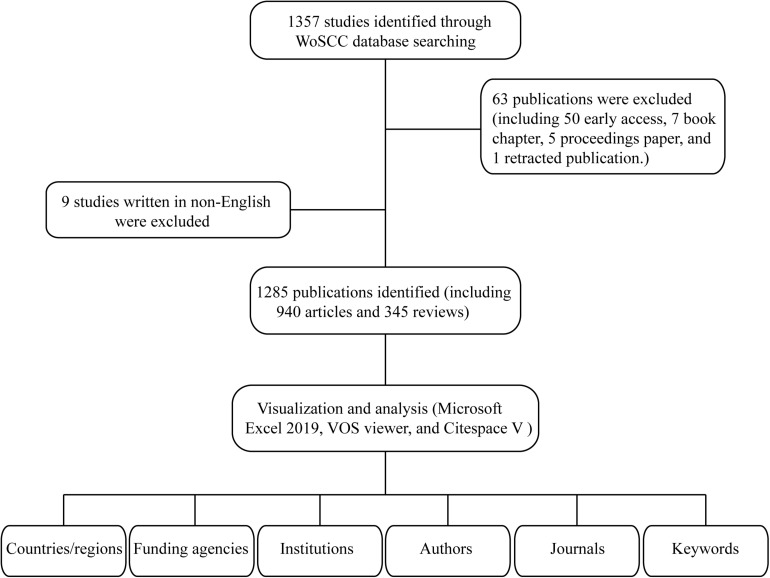
Flowchart for the selection of publications included in this study.

A systematic literature search was conducted by two independent authors in WoSCC for relevant publications and used the following search strategy: Topic: (ferroptosis) AND Language: (English). The first study pertaining to ferroptosis research that can be retrieved from WoSCC database was published by Dixon et al. (2012). Therefore, the timespan for data retrieval was set from 2012 to 2020. For manuscript types, only peer-reviewed original articles and reviews were included to ensure quality research, and all other source types were excluded (Figure 1).

### Data Collection

All results were searched on WoSCC using the formula above and exported together with full records including titles, authors, abstracts, and cited references in TXT format. The literature search was performed on a single day, November 12, 2020, in case of the possible biases introduced by update of the database.

### Data Extraction and Analysis

The TXT format files were imported to Microsoft Excel 2019 (Microsoft Corporation, Redmond, WA, United States) for quantitative and qualitative analysis. Different outputs were extracted independently by two authors, including annual research, countries/regions, funding agencies, source journals, institutions, authors, keywords, and citations. The number of annual publications and average citation of per publication were calculated. As indicators of the publications’ repercussion, we used the impact factor (IF) and Journal Citation Reports (JCR) category for the quality assessment of scientific information. IF is published annually by the Institute for Scientific Information in the JCR section of the SCI, which is the average number of citations of each article published in this journal during the period of the last 2 years. The JCR ascribes each of the scientific journals to their corresponding IF, and ranking them by specific areas, a marker of scientific “prestige.” In this study, we used the IF and category data published in the JCR of 2019.

Another indicator included in the present analysis was H-index (Hirsch, 2005), which is defined as one researcher has published the number of *h* articles, each of which has obtained at least a citation frequency of *h* times. It is often recognized as a measurement to evaluate the scientific output and academic status of a researcher and is also considered to be a useful indicator to evaluate both the productivity and impact of a country, institution, or journal. GraphPad Prism 8.0 (GraphPad Software Inc.) was also applied to analyze data and create graphs.

### Visualized Analysis

First, data files were imported into the free online analysis platform of literature metrology^1^ for preliminary bibliometric analysis. Several freeware visualization tools, such as VOS viewer, CiteSpace, Pajek, and BibExcel, also have been developed to help researchers create knowledge network maps, trace scientific developments, and identify emerging hotspots in a research field (Chen, 2004; Synnestvedt et al., 2005; van-Eck and Waltman, 2010). The three most frequently used methods are co-authorship, co-citation, and co-occurrence analysis. In this study, we have used countries/regions, institutions, journals, authors’ co-authorship and co-citation, and keyword co-occurrence analyses. In addition, countries/regions and institutions citation analysis were also performed.

Co-authorship analysis refers to the evaluation of the relationship among items through the number of co-authored documents. For example, author co-authorship analysis reveals cooperative relationship among authors, which may help new researchers to better understand existing partnerships and identify potential collaborators in a field.

Citation analysis captures the relationships between cited items. The relatedness of items is determined by the number of items that they cited each other. It was often performed to identify the key countries or institutions.

Co-citation analysis illustrates the relationship among items based on the number of times they are referenced together. The citations of an article can provide significant insight into what we currently know about a given research topic. The strength of co-citation relationship can help researchers identify the intellectual base and research frontiers within the field, important authors, and other bibliometric information.

Keyword co-occurrence analysis refers to the number of works where they occur together and weighted by the frequency of occurrence. If two keywords co-occur frequently in one publication, they may have a closer relationship to each other than other keywords, which may help researchers identify research hotspots and trends in a subject, and even inspire a new research idea.

#### Analysis With VOS Viewer

The java program VOS viewer^2^ (van-Eck and Waltman, 2010, Leiden University, Netherlands) was used for building network visualization maps. It is a software tool, which is commonly used for mapping and clustering of the scientific literature based on bibliometric data. In this study, this software was used for (i) countries/regions co-authorship and citation, (ii) institutions’ co-authorship and citation, (iii) authors’ co-authorship and co-citation, and (iv) keywords co-occurrence.

In VOS viewer maps, each node represents a different parameter such as countries/regions, institutions, or keywords. The size of the nodes is determined by the weight of the parameter, such as the number of publications, citation times or occurrence frequency. The higher the weight, the larger the nodes. The color of the nodes and lines is determined by the cluster they belong. The line between the nodes represents links. And the strength of the links was assessed by the indicator of total link strength (TLS), which can be scaled up to reflect the total co-authorship and co-citation link strength between countries, institutions, or authors.

#### Analysis With CiteSpace

In this scientometric study, CiteSpace V^3^ (Version 5.7 R2, Chen et al., 2014, Drexel University, United States) was used to (i) create the dual-map overlay of journals to capture the relationship between citing journals and cited journals, (ii) detect a citation-burst analysis of references and keywords, and (iii) generate the keywords network visualizations map from the time zone view.

For all network visualizations, parameters of CiteSpace were set as follows: time slicing (2012–2020), years per slice (1 year), term source (title, abstract, author keyword, keyword plus), node type (choose one parameter at a time such as cited journal, reference, or keywords), selection criteria (top 50 or 100), pruning (minimum spanning tree, pruning sliced networks), and visualization (time zone view). A more detailed description of the software, utilization skills, and options can be found in the CiteSpace manual.

### Research Ethics

Ethical approval was not required, as the data used in this article were downloaded from the public databases and did not involve interaction with human participants.

## Results

### Publication Outputs and Citation Trend

**FIGURE 2:**
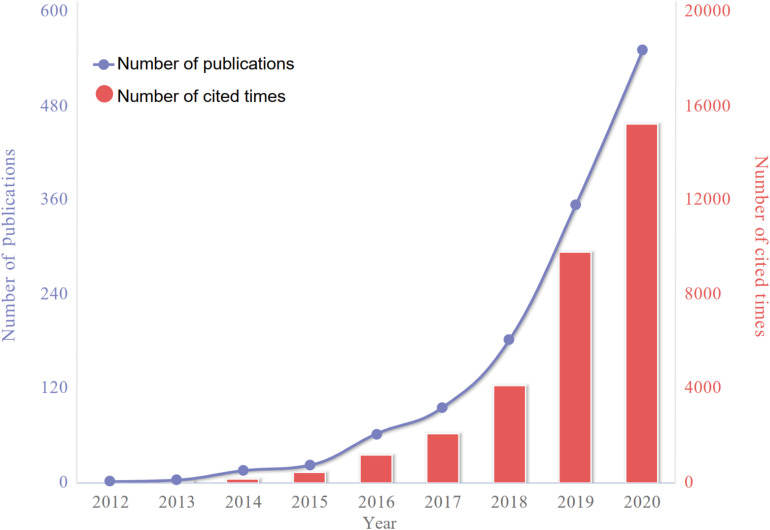
The number of articles published annually and the summed total citations of annual articles related to ferroptosis have been steadily increasing from 2012 to 2020.

A total of 1,285 ferroptosis-related publications including 940 articles and 345 reviews were extracted from WoSCC bibliographic database. The number of publications per year before 2017 was fewer than 100. After 2018, the annual number of relevant publications started increasing rapidly. And up to date (November 12, 2020), more than 500 articles have been published in 2020. The sum number of citations was 33,145, and the average number of citations per item was 25.8 times (Figure 2).

### Contributions of Countries/Regions and Funding Agencies

**FIGURE 3:**
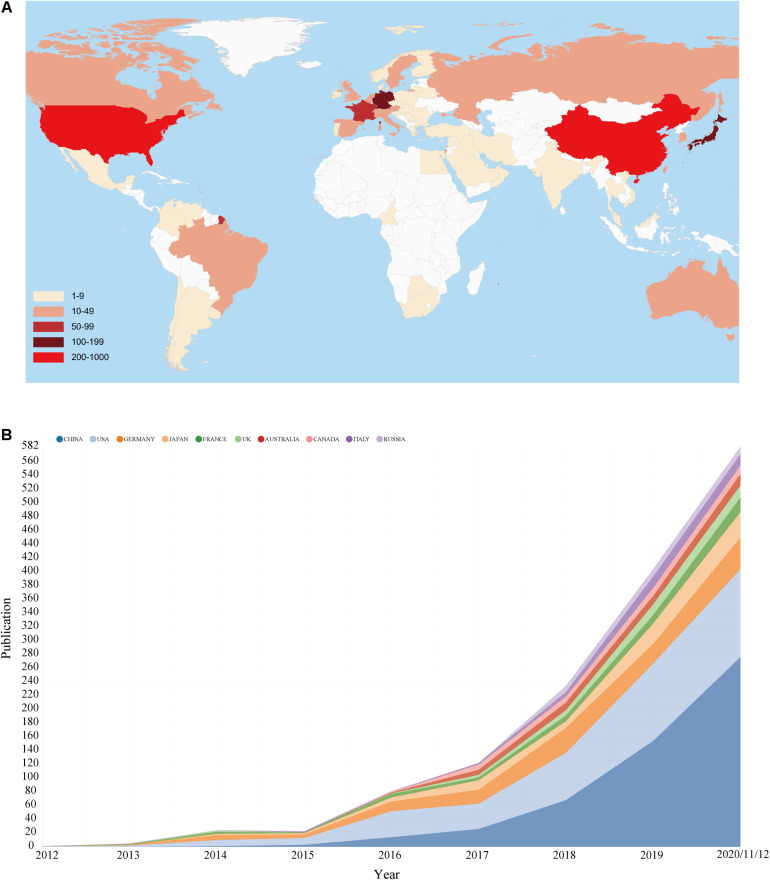
**(A)** Geographical distribution map of global publications related to ferroptosis. **(B)** The changing trend of the annual publication counts in the top 10 countries/regions from 2012 to 2020.

Geographical distribution map of global productivity revealed that articles on ferroptosis had been mainly published from Asia, North American, and European countries (Figure 3A). The top 10 most prolific countries/regions in ferroptosis publications are listed in Table 1, and changing trend of the annual publication counts in these countries/regions from 2012 to 2020 is displayed in Figure 3B. The leading country was China, which took up 43.74% (562/1,285) of the total number of publications, followed by the United States (418, 32.53%) and Germany (157, 12.22%). The United States was cited the most (20,980 times) and achieved the highest H-index (70).

**TABLE 1:** The top 10 productive countries with publications concerning ferroptosis.

Rank	Countries	Article counts	Percentage (n/1,285)	H-index	TLS	Total citations	Average citation per article
1	China	562	43.74%	46	16,494	9,681	17.23
2	United States	418	32.53%	70	18,742	20,980	50.19
3	Germany	157	12.22%	44	8,289	8,848	56.36
4	Japan	105	8.17%	24	4,056	3,979	37.9
5	France	56	4.36%	24	2,588	2,736	48.86
6	Australia	48	3.74%	21	2,302	2,777	57.85
7	Canada	43	3.35%	19	1,896	1,917	44.58
8	England	43	3.35%	16	1,761	2,848	66.23
9	Italy	41	3.19%	11	1,207	1,415	34.51
10	Russia	34	2.65%	13	1,405	1,487	43.74

**FIGURE 4:**
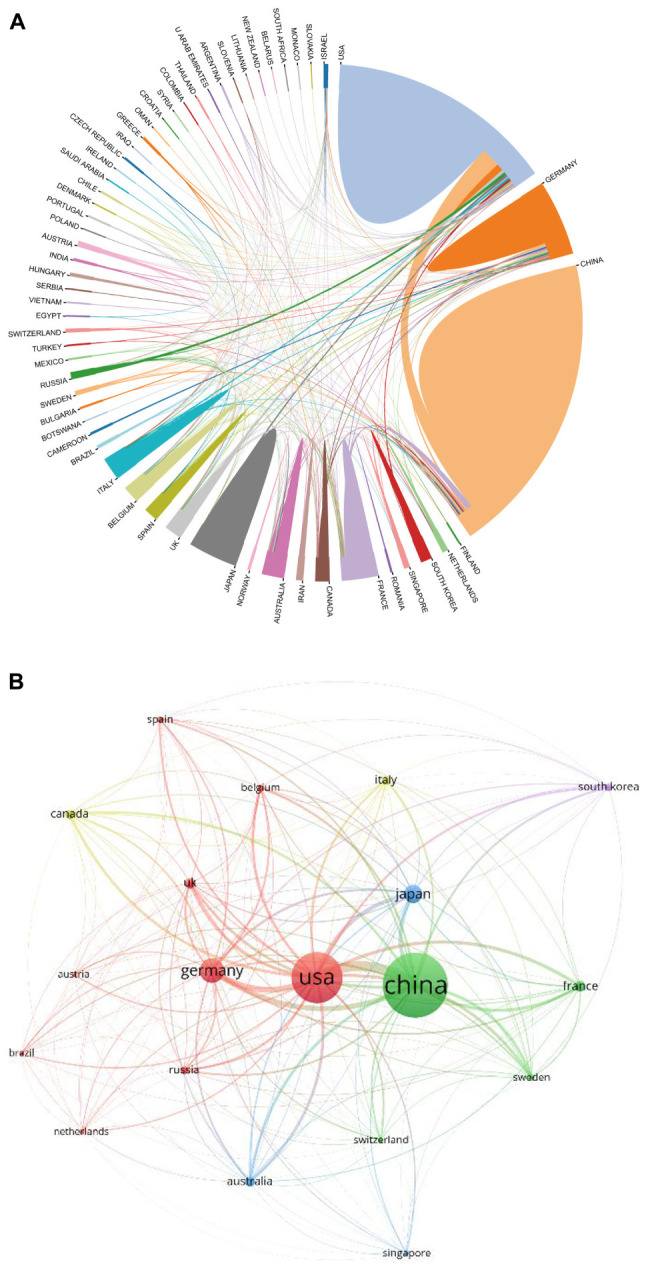
**(A)** The cross-country/region collaborations visualization map. **(B)** The countries/regions citation network visualization map generated by VOS viewer software.

Supplementary Table 1 lists the world’s top 10 funding agencies for the output of ferroptosis research. Among them, funding agencies from China and the United States sponsored the highest number of studies. The cross-country collaboration map indicated that cooperation among countries/regions was relatively close (Figure 4A). China collaborated most closely with the United States and France. The United States also cooperated frequently with Germany. The citation relationship among different countries/regions was visualized in a citation network map (Figure 4B). Only countries/regions with a minimum of 10 publications were visualized. Of the 19 countries/regions that met this threshold, the top three with the largest TLS were the United States, China, and Germany.

### Contributions of Institutions

A total of 1,453 institutions contributed to the publications on ferroptosis. The top 10 productive institutions ranked by the numbers of publications are presented in Table 2. The vast majority of the scientific research institutions were from China and the United States. Columbia University and University Pittsburgh contributed the most publications, followed by Guangzhou Medical University and Chinese ACDA of Sciences. Columbia University had the highest value of H-index and the average number of citations per publication.

**TABLE 2:** The top 10 productive institutions ranked by the numbers of publications.

Rank	Institutions	Countries	Article counts	H-index	TLS	Total citations	Average citation per article
1	Columbia University	United States	59	38	6,738	10,113	171.41
2	University of Pittsburgh	United States	59	30	5,335	5,572	94.44
3	Guangzhou Medical University	China	42	22	4,511	3,822	91
4	Chinese Academy of Sciences	China	33	18	857	1,769	53.61
5	Zhejiang University	China	33	8	1,231	499	15.12
6	Central South University	China	29	16	1,775	1,427	49.21
7	Stanford University	United States	29	18	2,133	3,484	120.14
8	Helmholtz Zentrum München	Germany	28	20	2,927	2,655	94.82
9	Jilin University	China	25	11	1,541	639	25.56
10	Shanghai Jiao Tong University	China	25	7	981	468	18.72

**FIGURE 5:**
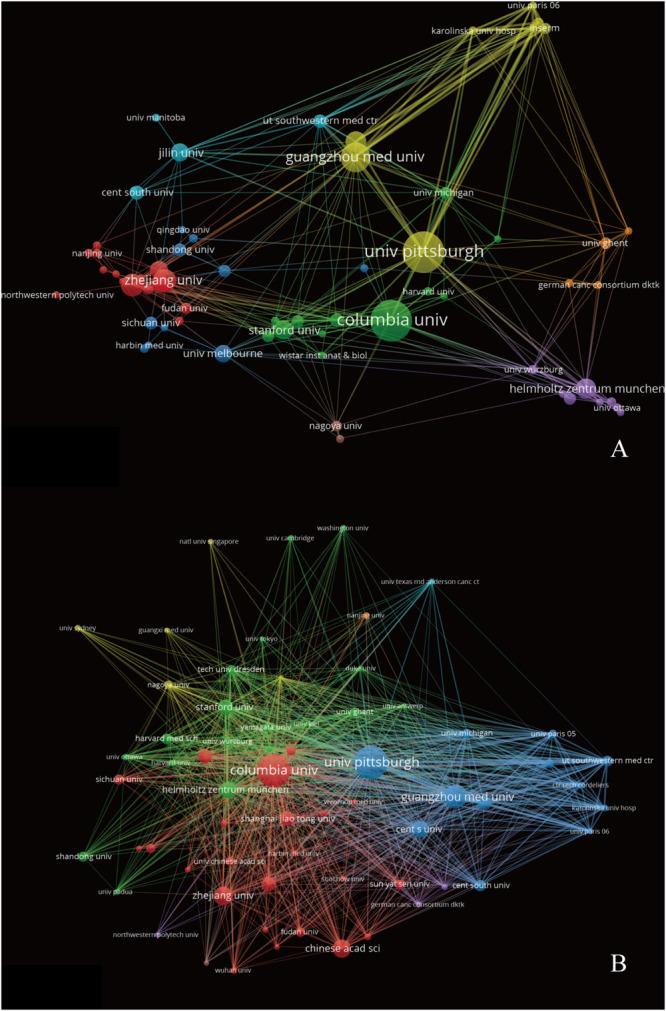
The institutions’ collaboration **(A)** and citation **(B)** network visualization map generated by VOS viewer software.

The network visualization map for institutions’ collaboration and citation analysis was generated by VOS viewer. Institutions with a minimum of 10 publications were visualized. As shown in Figure 5A, the collaboration map had 64 nodes and 313 links. The 64 institutions formed eight clusters with different colors, and there was active collaboration between the institutions, especially among the institutions in the same cluster. As for the citation analysis shown in Figure 5B, there were 64 nodes and 1,840 links in the network map. The top three institutions with largest TLS are listed as follows: Columbia University (TLS = 6,738), University Pittsburgh (TLS = 5,335), and Guangzhou Medical University (TLS = 4,511).

### Authors and Co-cited Authors

**FIGURE 6:**
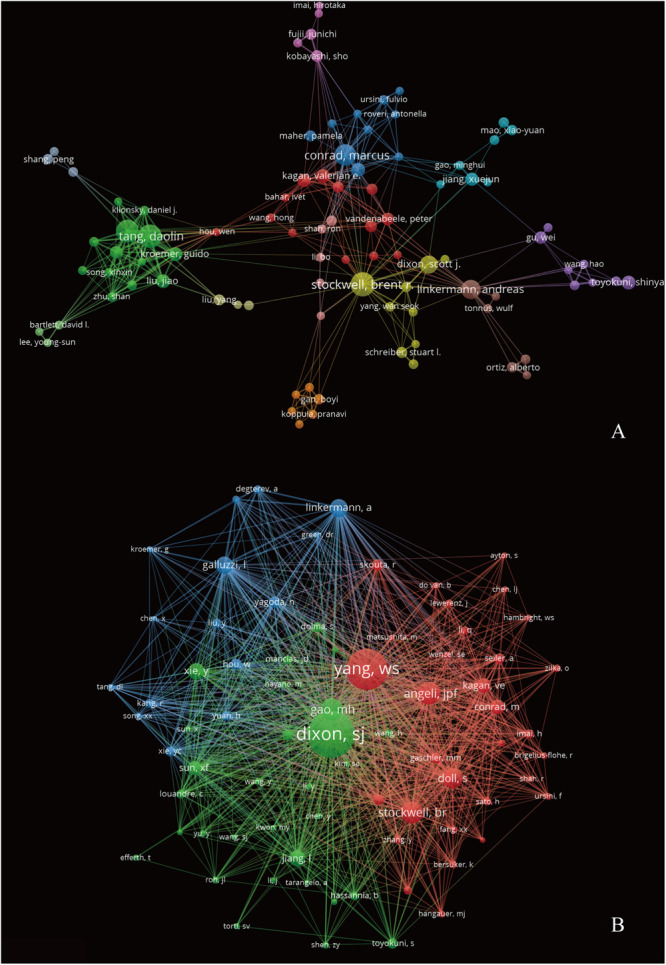
The authors’ collaboration **(A)** and co-citation **(B)** network visualization map generated by VOS viewer software.

A total of 6,958 authors were obtained in the 1,285 publications. The top 10 most productive authors contributed 177 articles (13.77%) on ferroptosis research. B. R. Stockwell from Columbia University contributed the most articles (47 articles), followed by D. L. Tang and R. Kang from University of Texas Southwestern Medical Center at Dallas, with 38 and 37 publications, respectively (Table 3). From the cooperative network map of authors, D. L. Tang, R. Kang, M. Conrad, and B. R. Stockwell were located at a central position of the cooperating clusters. There was active collaboration among the productive authors (Figure 6A). The co-citation network map contained 76 nodes, 2,847 links and 3 clusters (Figure 6B). The top three authors with largest TLS were S. J. Dixon (TLS = 28,799), W. S. Yang (TLS = 27,302), and J. P. Friedmann-Angeli (TLS = 13,394).

**TABLE 3:** The top 10 most productive authors in ferroptosis publications.

Rank	Author	Count	H-index	Total citations	Countries	Institutions
1	B. R. Stockwell	47	33	9,552	United States	Dept Biol Sci/Chem, Columbia Univ
2	D. L. Tang	38	20	3,226	United States	Dept Surg, Univ Texas Southwestern Med Ctr Dallas
3	R. Kang	37	20	2,261	United States	Dept Surg, Univ Texas Southwestern Med Ctr Dallas
4	M. Conrad	36	26	4,762	Germany	Inst Metab & Cell Death, Helmholtz Zentrum Munchen
5	A. Linkermann	28	20	3,945	Germany	Dept Nephrol, Tech Univ Dresden
6	S. J. Dixon	25	18	5,629	United States	Dept Biol, Stanford Univ
7	J. Liu	24	10	337	China	Affiliated Hosp 3, Guangzhou Med Univ
8	Y. Liu	23	8	219	China	Affiliated Hosp 3, Guangzhou Med Univ
9	V. E. Kagan	22	14	2,472	United States	Dept Environm & Occupat Hlth, Univ Pittsburgh
10	Y. Zhang	22	8	172	China	Tianjin Key Lab Spine & Spinal Cord, Tianjin Med Univ

### Journals and Co-cited Journals

A total of 476 scholarly journals published articles on ferroptosis research. The top 10 active journals published 246 articles, accounting for 19.14% of all the publications (Table 4 and Supplementary Figure 1). *Cell Death Disease* (IF 2019, 6.304) published the highest number of articles (42 publications, 3.27%), followed by *Biochemical and Biophysical Research Communications* (IF 2019, 2.985) and *Free Radical Biology and Medicine* (IF 2019, 6.17). Among the top 10 journals, four were from England, three from the United States, two from Switzerland, and one from the Netherlands. *Free Radical Biology and Medicine* has the highest value of H-index (18), and *Cell Death and Differentiation* has the largest number of total citations (1857 times) and highest IF (IF 2019, 10.717).

**TABLE 4:** The top 10 journals of ferroptosis research ranked by publication number.

Rank	Journal Title	Country	Count	IF (2019)	Quartile in category (2019)	H-index	Total citations
1	*Cell Death Disease*	England	42	6.304	Q1	12	526
2	*Biochemical and Biophysical Research Communications*	United States	36	2.985	Q2	12	848
3	*Free Radical Biology and Medicine*	United States	31	6.17	Q1	18	1,034
4	*Redox Biology*	Netherlands	27	9.986	Q1	11	678
5	*Cell Death and Differentiation*	England	22	10.717	Q1	12	1,857
6	*International Journal of Molecular Sciences*	Switzerland	21	4.556	Q1/Q2	7	170
7	*Oxidative Medicine and Cellular Longevity*	England	20	5.076	Q2	6	172
8	*Scientific Reports*	England	17	3.998	Q1	7	220
9	*Cell Chemical Biology*	United States	15	7.739	Q1	6	209
10	*Frontiers in Neuroscience*	Switzerland	15	3.707	Q2	7	237

**FIGURE 7:**
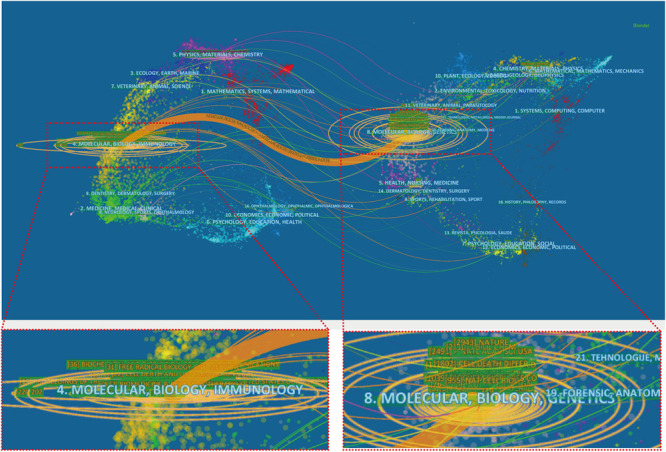
A dual-map overlay of the journals on ferroptosis research generated by using CiteSpace software.

Figure 7 presents a dual-map overlay of the number of publications with reference to the focus of the journals. The labels on the map represent different research subjects covered by all the journals, which can reveal trends of the scientific portfolio in the overall visualization. The left half of the map represents the citing journals, whereas the right half represents the cited journals. Different colored lines represent different paths of references, where each line starts from the citing map and ends at the cited map. The width of the connecting paths is proportional to the frequency of *z*-score-scale citation. Overall, there was one main citation path in the current map. The published articles targeted journals in the fields of molecular, biology, and immunology, whereas the most cited articles were published in the journals of molecular, biology, and genetics.

### References and Co-cited References

**FIGURE 8:**
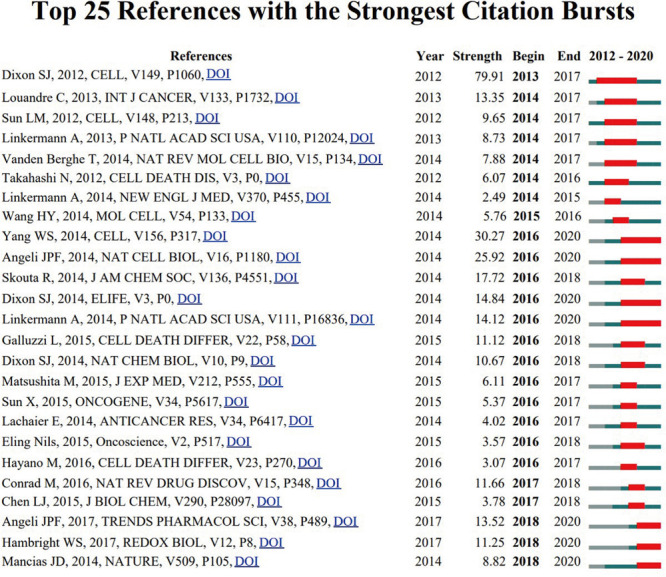
The top 25 references with the strongest citation bursts during 2012 to 2020.

The top 10 ferroptosis-related original articles with the most citations are shown in Table 5. And the top 10 review articles of this field with the most citations are also summarized in Supplementary Table 2. *Cell* and *Nature Chemical Biology* have a tremendous scientific impact on researchers and academics in the field, and half of the top 10 highly cited original articles were published in these journals. All the top 10 references were co-cited more than 340 times. The study by Dixon et al. (2012), published in *Cell*, was the most cited article with 1,958 times up to now. CiteSpace software was used to explore the references with strong citation bursts, and 25 references with the strongest citation bursts were identified. “References with citation bursts” means that the corresponding studies have been frequently cited within a certain period. In Figure 8, the blue line segment represents the time interval, and the red line segment represents the active time. References with citation bursts first emerged in 2013, and the burst was due to a publication in 2012. Approximately 80% of the references had citation bursts between 2014 and 2016. The most recent reference with a citation burst was observed in 2018, and the burst is still ongoing.

**TABLE 5:** The top 10 ferroptosis-related original articles with the most citations (up to March 9, 2021).

Title	First author	Journal	Year	Citations	Main conclusion
Ferroptosis: An Iron-Dependent Form of Nonapoptotic Cell Death	S. J. Dixon	*Cell*	2012	1,958	They found that RAS-selective lethal small molecule erastin could selectively induce a unique nonapoptotic form of cell death in cancer cells, which could also be inhibited by iron chelators or lipophilic antioxidants like ferrostatin-1, and first named this iron-dependent form of programmed cell death as ferroptosis.
Regulation of Ferroptotic Cancer Cell Death by GPX4	W. S. Yang	*Cell*	2014	1,021	They determined that the second ferroptosis inducer, RSL3, could directly inhibit GPX4 activity. Gpx4 is a central regulator of ferroptosis, which could be induced in mouse tumor xenografts.
Inactivation of the Ferroptosis Regulator Gpx4 Triggers Acute Renal Failure in Mice	J. P. Friedmann Angeli	*Nature Cell Biology*	2014	638	They provided direct genetic evidence that the knockout of Gpx4 caused cell death, which was associated with ferroptosis-related pathological types.
Ferroptosis as a p53-Mediated Activity During Tumour Suppression	L. Jiang	*Nature*	2015	549	They found that that p53 inhibits cystine uptake and sensitizes cells to ferroptosis by repressing expression of SLC7A11 and claimed that they uncovered a new mode of tumor suppression based on p53 regulation of ferroptosis.
ACSL4 Dictates Ferroptosis Sensitivity by Shaping Cellular Lipid Composition	S. Doll	*Nature Chemical Biology*	2017	422	They revealed that inhibition of ACSL4 was effective in protecting against RSL3-induced cell death, suggesting that ACSL4 inhibitor was a feasible treatment for the prevention of ferroptosis-related diseases.
Pharmacological Inhibition of Cystine-Glutamate Exchange Induces Endoplasmic Reticulum Stress and Ferroptosis	S. J. Dixon	*Elife*	2014	398	They reported that the small molecule erastin was a very effective inhibitor of system xc-, and the anticancer drug sorafenib could inhibit system xc-, which was different with other drugs in the same class as sorafenib.
Oxidized Arachidonic and Adrenic PEs Navigate Cells to Ferroptosis	V. E. Kagan	*Nature Chemical Biology*	2017	394	Suppression of AA or AdA esterification into PE could act as a specific antiferroptotic pathway. Vitamin E was shown to regulate ferroptosis via LOX inhibition.
Glutaminolysis and Transferrin Regulate Ferroptosis	M. Gao	*Molecular Cell*	2015	371	Transferrin and amino acid glutamine related to iron homeostasis were identified as the inducers of ferroptosis, and they also reported that cell surface transferrin receptor and glutaminolysis played crucial roles in the ferroptosis process.
Activation of the p62-Keap1-NRF2 Pathway Protects against Ferroptosis in Hepatocellular Carcinoma Cells	X. Sun	*Hepatology*	2016	345	This study demonstrated that the activation of the NRF2 pathway protected against ferroptosis in hepatocellular carcinoma cells. The inhibition of NRF2 expression/activity in hepatocellular carcinoma cells could increase the anticancer activity of erastin and sorafenib.
Synchronized Renal Tubular Cell Death Involves Ferroptosis	A. Linkermann	*Proceedings of the National Academy of Sciences of the United States of America*	2014	341	They demonstrated that ferroptosis directly caused synchronized necrosis of renal tubules and generated a novel third-generation ferrostatin called 16–86, which was more stable and potent in plasma and liver microsomes compared with Fer-1.

### Keywords Analysis of Research Hotspots

Keywords were extracted from titles and abstracts of all the 1,285 publications. A density visualization map was generated for keywords with the co-occurrence greater than 50 times, which includes 99 keywords in the map (Supplementary Figure 2 and Supplementary Table 3).

**FIGURE 9:**
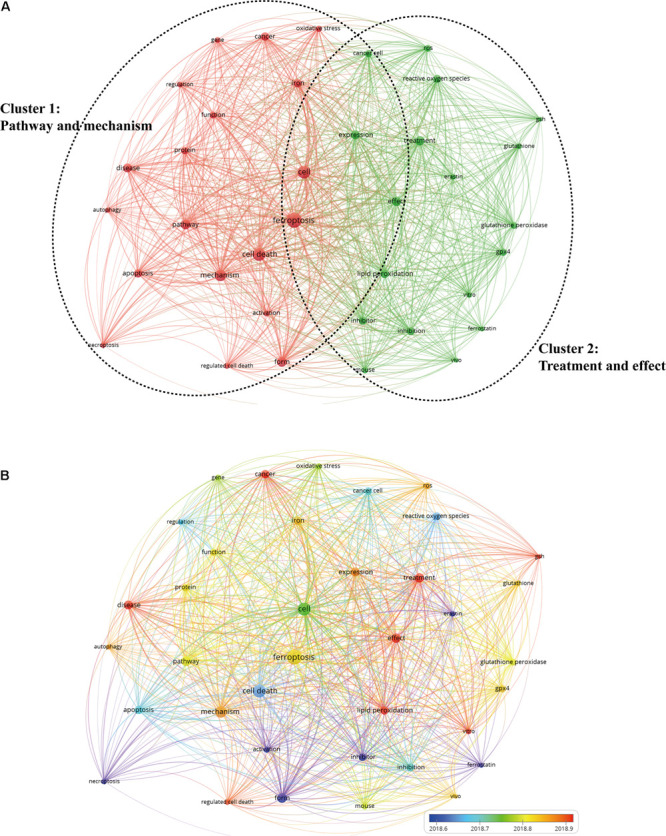
The network visualization map of keywords by VOS viewer. **(A)** Map of keywords clustering showed 37 keywords with a minimum of 100 occurrences and divided into two clusters. **(B)** Different colors were applied for each keyword based on their average appearance time in the overlay visualization map. Blue color represented the keywords that appeared relatively earlier than those in yellow and red upon time course.

Clustering analysis of high-frequency keywords (more than 100 times) was performed. As shown in Figure 9, there were 37 nodes and 666 links in the network map after filtering out some common keywords. All the keywords could be classified into two clusters: cluster 1 (pathway and mechanism of ferroptosis; Figure 9A, left, in red), and cluster 2 (treatment and effect; Figure 9A, right, in green).

Cluster 1 was the larger cluster. The prominent keywords were ferroptosis (1,014 times), cell death (743 times), mechanism (586 times), and pathway (467 times). As for the cluster 2, the primary keywords were cell (739 times), effect (421 times), treatment (396 times), and inhibitor (283 times). In Figure 9B, VOS viewer could mark keywords included in the overlay visualization map with different colors based on their average appearing year. The blue color represented the keywords that appeared relatively earlier than those in yellow and red upon the time course. As can be seen from Figure 9B, a trend of balanced development existed in both the clusters over the last few years. Large numbers of research hotspots related to ferroptosis have emerged in recent years, which indicates that the field is evolving at a tremendously fast pace.

**FIGURE 10:**
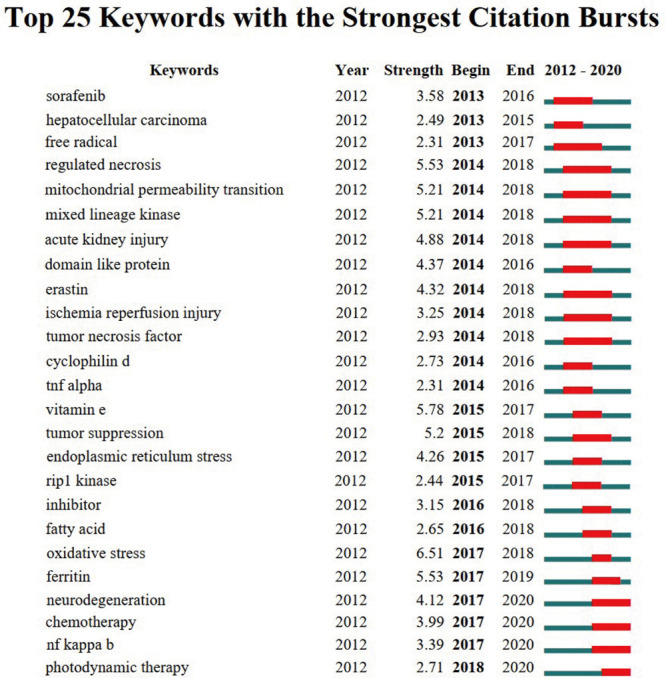
The top 25 keywords with the strongest citation bursts of publications on ferroptosis research.

The keywords with strong burst strength were another important indicator to reflect the research hotspots, frontiers, and emerging trends over time (Figure 10). Most notably, the citation burst time of keywords including “neurodegeneration” (2017–2020), “chemotherapy” (2017–2020), “NF-kappa B” (2017–2020), and “photodynamic therapy” (2018–2020) has continued to 2020, and the bursts are still ongoing, indicating that these research directions have been getting great attention in recent years and also have the potential to become new research hotspots in the future.

## Discussion

In such an era of information explosion, it is getting ever more difficult to remain in the top and grasp the latest research results. Knowledge management is often one of the concerned issues for scientific staff (Tijssen and Winnink, 2016). In this study, we aimed to provide an innovative way to manage and visualize the knowledge structures of a research area by employing a bibliometric analysis. Our findings provide an updated analysis of the global scientific output related to ferroptosis research from 2012 to 2020.

As shown in Figure 2, a huge increase in the number of global publications on ferroptosis research was validated from 2012 to 2020. Prior to 2017, global publications on ferroptosis research exhibited a stepwise growth, whereas the past 3 years has witnessed a dramatic growth. Accordingly, it seems possible to speculate that this field is about to enter its golden period in the next few years.

In terms of countries/regions analysis, China and the United States were the most productive countries in this field, which had an overwhelmingly higher number of publications than other countries/regions. Of course, this is inseparable from the local funding agencies. Among top 10 funding agencies, 70% were from China and the United States.

Also notably, although China surpasses the United States in regard to the number of publications, the United States maintained the dominant position in this field with the most citations and the highest H-index. As we can see from the citation network map, the United States had the largest TLS, which indicated that the impact of articles published by the United States might be higher. Therefore, from a research quality point of view, the United States was actually dominated in this field. Moreover, it is noteworthy that policymakers of China have noticed this issue and also developed a series of measures to improve the quality of academic articles.

In the list of top 10 productive institutions, with the exception of three institutions from the United States and one from Germany, the remaining six institutions were all from China. This may be the reason for China contributing to the most publications related to ferroptosis research. Meanwhile, these results also suggest that establishing topnotch research institutions is the pivotal basis to elevate the national academic status.

Additionally, on the list of the top 10 most prolific authors, five were from the United States, three were from China, and the other two were from Germany. B. R. Stockwell from Columbia University, as well as D. L. Tang and R. Kang from the University of Texas Southwestern Medical Center at Dallas, contributed the most publications and played important leadership roles in the field. As a result, further significant publications related to ferroptosis are more likely to publish these authors and their teams. And top scholars from the top institutions can be good choices for research collaboration as they may have priority access to grant/funding support.

Regarding the top journals, those listed in Table 4, such as *Cell Death Disease*, *Biochemical and Biophysical Research Communications*, *Free Radical Biology and Medicine*, *Redox Biology*, and *Cell Death and Differentiation* may be the core journals of ferroptosis research publication. Further manuscripts can be guided for submission to these journals. Among the top 10 journals, only one of the journals, that is, *Cell Death and Differentiation* (IF2019, 10.717), had an IF greater than 10.0. Five of the journals, including *Cell Death Disease* (IF2019, 6.304), *Free Radical Biology and Medicine* (IF2019, 6.17), *Redox Biology* (IF2019, 9.986), *Oxidative Medicine and Cellular Longevity* (IF2019, 5.076), and *Cell Chemical Biology* (IF2019, 7.739), had an IF between 5.0 and 10.0. Overall, it is a challenge to publish articles related to ferroptosis in high-IF journals.

“References with citation bursts” means that the corresponding studies have been frequently cited within a certain period. This also indicates that the publications have attracted considerable attention from the scientific community and could reflect dynamic changes and hotspots in ferroptosis research to some extent. The earliest burst beginning from 2013 was due to the article published in 2012, and the burst lasted for 5 years. This study defined a unique iron-dependent form of programmed cell death and named it *ferroptosis*, which marked the beginning of a new research field (Dixon et al., 2012).

Approximately 80% of the references had citation bursts between 2014 and 2016. Remarkably, the burst of four studies is still ongoing. Of these, Yang et al. (2014) found that Gpx4 was a central regulator of ferroptosis, which could be induced in mouse tumor xenografts. Friedmann-Angeli et al. (2014) further provided direct genetic evidence that Gpx4 knockout caused cell death in a pathologically relevant form of ferroptosis and also confirmed that glutathione/Gpx4 axis played an important role in preventing lipid oxidation–induced acute renal failure and associated death. Another study by Dixon et al. (2014) reported that the erastin was a potent, selective inhibitor of system xc (–). Sorafenib, which was a clinically approved anticancer drug, could inhibit system xc (–) function and trigger ER stress and ferroptosis. While Linkermann et al. (2014) explored that ferroptosis could directly induce synchronized necrosis of renal tubules. They also generated a novel third-generation ferrostatin termed 16–86, which was confirmed to be more stable and potent than the first-in-class compound (ferrostatin-1). More remarkably, the most recent strongest burst has begun since 2018, and the burst remains ongoing. This is mainly associated with three articles published in *Trends Pharmacol Sci* by Angeli et al. (2017), *Redox Biol* by Hambright et al. (2017), and in *Nature* by Mancias et al. (2014), which deserve further attention.

In bibliometrics, analysis of frequently appearing keywords can also reveal the changing trends and major topics, critical for understanding the development of this field. As shown in keywords clustering map in Figure 9, it was observed that all the keywords on ferroptosis research could separate into two clusters. Cluster 1 focused primarily on the pathway and mechanism of ferroptosis, and the prominent keywords were ferroptosis, cell death, mechanism, and pathway. While cluster 2 was mainly about treatment and effect, the primary keywords were cell, effect, treatment, and inhibitor. As a newly emerging and rapidly evolving field, this result was in accordance with the development law of a new discipline. Once the basic knowledge such as molecular mechanism and regulation of ferroptosis is recognized, a spectrum of potential clinical applications, including drug discovery and development, will follow. For application studies, ferroptosis pathways have gained attention as novel therapeutic targets for treatment of many diseases, in particular for cancers and neurodegenerative diseases (Chen et al., 2015; Liang et al., 2019; Lelièvre et al., 2020; Reichert et al., 2020; Sun et al., 2020). Therefore, parallel to the mechanism studies of ferroptosis, further studies should focus on clinical application and transformation research.

Keywords with the strongest citation bursts can provide a reasonable prediction of frontiers in ferroptosis research. In this instance, CiteSpace V mainly captured four research frontiers as follows: “neurodegeneration,” “chemotherapy,” “NF-kappa B,” and “photodynamic therapy.”

### (i) Neurodegeneration

Brain is one of the main target organs known to be affected by the disruption of iron homeostatic balance (Weiland et al., 2019; Tang et al., 2019). Growing evidence implicates ferroptosis participating in neurodegenerative diseases including Alzheimer disease (AD), Parkinson disease (PD), and Huntington disease (HD). AD is considered a common aging-related neurodegenerative disorder characterized by inflammation of the nerve cells, oxidative damage, and the formation of β-amyloid plaques (Reichert et al., 2020). Previous studies have demonstrated the iron accumulation in the cerebral cortex was associated with the development of AD. Recently, one study observed that a Gpx4 knockout mouse exhibited significant deficits in spatial learning and memory function, and markers related to ferroptosis, such as elevated lipid peroxidation, and augmented neuroinflammation were also detected (Hambright et al., 2017). Some other studies also have found evidence in support for a connection between AD and ferroptosis, and Gpx4 could protect cortical neurons from oxidative stress and amyloid toxicity (Zhang et al., 2018; Huang et al., 2020). However, whether ferroptosis is the cause or consequence of AD is still unclear.

In addition, iron accumulation and oxidative damages are also common features in PD and HD (Masaldan et al., 2019; Mi et al., 2019; Chen X. et al., 2020). PD is recognized as the second most common neurodegenerative disorder behind AD. Current therapy is primarily based on a dopamine replacement strategy using the dopamine precursor levodopa. Previous studies on the pathogenesis of PD showed that iron was a cofactor for tyrosine hydroxylase, and the iron level in substantia nigra could directly interfere with the expression of dopamine (Zucca et al., 2017). A number of reports have demonstrated that iron accumulation and oxidative damages may trigger and accelerate AD progression (Jiang et al., 2016; Mu et al., 2020). In the meantime, treatments with antioxidants and iron chelators (such as VK-28, EGCG, and M30) have been shown to be neuroprotective in PD (Moreau et al., 2018; Kuang et al., 2020; Mu et al., 2020).

As the candidates for neuronal cell death pathways, despite ferroptosis, have only been investigated in neurodegenerative disorders for only a few years, it has quickly become one of hot research directions for involvement of this pathway in these diseases (Doll et al., 2017; Li et al., 2020). Therefore, future research should not only focus on the evidence in humans that ferroptotic signaling pathway is definitely involved in the neurodegenerative disorders, but also to find promising targets for pharmacological intervention (Doll et al., 2017; Kagan et al., 2017). For example, as a key role of cytoplasmic peroxidation inhibiting protein, Gpx4 has been regarded as a promising therapeutic target of neurodegenerative diseases (Cong et al., 2019). And targeting iron, including deferoxamine, deferiprone, and deferasirox, to decrease iron overaccumulation is another promising treatment strategy. Predictably, substantial effort and funds will be devoted to the development of novel drugs in the future.

### (ii) Chemotherapy

In addition to the neurodegenerative diseases mentioned previously, accumulative data suggest that the cancer cells require a substantial iron supply for their rapid proliferation than the normal cells, which renders tumor cells more sensitive to ferroptotic process (Jiang et al., 2015; Lelièvre et al., 2020; Lin and Chi, 2020). Increasing studies have been devoted to mining the therapeutic potentials of inducing ferroptotic cell death in several types of human tumors (summarized in Supplementary Table 4).

Chemotherapy is one of the main therapeutic options for the treatment of malignant tumors. Most chemotherapeutic agents rely on the induction of apoptotic death pathways because of their cytotoxic effect to kill tumor cells. However, chemoresistance is one of the key obstacles to the clinical application of chemotherapeutic drugs. Recently, several clinically important chemotherapy drugs or drugs have a strong potential for clinical application and were found to induce ferroptotic cancer cell death (Xu et al., 2019). Besides this, as ferroptosis is iron-dependent form of cell death, which is totally different from apoptosis, necrosis, or pyroptosis, thus the combinations with ferroptotic reagents have the opportunity to circumvent the chemoresistance of apoptosis-inducing chemotherapy drugs and improve the outcomes (Friedmann-Angeli et al., 2019; Liang et al., 2019). At present, growing preclinical evidence suggests that ferroptosis-induced cell death was able to prevent emergence of acquired resistance in several chemotherapy drugs, such as lapatinib, trametinib, and erlotinib, and tumor cells selected for drug resistance exhibit the EMT phenotype (characterized by downregulation of epithelial markers and loss of intercellular junctions) (Sun et al., 2016; Friedmann-Angeli et al., 2019; Chen et al., 2021). Based on current data, targeting ferroptosis is a promising approach to overcome tumor resistance and will certainly be one of the most exciting research hotspots in cancer chemotherapy.

### (iii) Nuclear Factor κB

The nuclear factor κB (NF-κB) comprises a family of inducible transcription factors and has a central role in regulating the expression of a wide variety of genes associated with immune and inflammatory response (Li and Verma, 2002). Studies have affirmed agents that inhibited this pathway (e.g., aspirin and glucocorticoids) could reduce the inflammatory response. In recent studies, the interaction of ferroptosis with the NF-κB pathway in regulating inflammatory responses has attracted the attention of scholars. Li et al. (2018) demonstrated that Gpx4 activator can suppress inflammatory conditions through the inhibition of the NF-κB pathway. And other researchers also discovered that erastin had similar roles in inflammatory response by suppressing the NF-κB signaling pathway, resulting in inhibition of sepsis development (Oh et al., 2019).

Additionally, there is growing evidence suggesting that NF-κB is involved in the regulation of ER stress signaling and ferroptosis process (Chen P.H. et al., 2020; Xu M. et al., 2020). Xu M. et al. (2020) have investigated the role of ferroptosis in ulcerative colitis. And their findings indicated that ferroptosis contributed to intestinal epithelial cell death through ER stress signaling pathway in ulcerative colitis. And phosphorylated–NF-κBp65 could alleviate intestinal epithelial cells ferroptosis via suppressing ER stress. From the above studies, ferroptosis may play an important role in multiple known and unknown inflammatory diseases. And NF-κB signaling pathway is a potential therapeutic target for ferroptosis-mediated inflammatory diseases.

### (iv) Photodynamic Therapy

Photodynamic therapy (PDT) is a minimally invasive treatment modality that requires the combination of a photosensitizer, light of a specific wavelength, and tissue oxygen (Lee et al., 2011; Kwiatkowski et al., 2018). After accumulating in target tissues, the photosensitizer is activated by a light source of a specific wavelength for efficient generation of reactive oxygen species (ROS), predominantly singlet oxygen (^1^O_2_), and subsequently yields cell damage and death (Lee et al., 2011). It has emerged as a promising and clinically approved strategy in the treatment of various cancers (Agostinis et al., 2011). However, the rapid oxygen consumption during the process of PDT may induce adverse domino effects, which further aggravate the state of oxygen deprivation around the tumors and thus inhibit the efficiency of PDT process, resulting in unsatisfactory efficacy (Wang et al., 2018). Therefore, how to increase the concentration of ROS especially ^1^O_2_ in the tumor microenvironment remains an important bottleneck for PDT to be addressed.

Of note, iron plays a key role in ferroptosis, which could catalyze the formation of ROS through Fenton reaction, defined as the reaction of ferrous iron and hydrogen peroxide (He et al., 2020). Several studies have found that significant tumor suppression could be achieved by inducing Fenton reaction–dependent ferroptosis (Shen et al., 2018; Yu et al., 2020). Considering that the essential ferroptosis process is ROS accumulation, which is just exactly needed during PDT process, some scholars have begun to put forward the concept of ferroptosis-promoted PDT. The results proved that ferroptosis-promoted PDT approach dramatically enhanced anticancer efficacy by promoting ROS production and relieving hypoxia, revealing the promising prospects of combining ferroptosis and PDT therapy (Zhu et al., 2019; Xu T. et al., 2020; Mishchenko et al., 2021). Beyond this, further studies have found that highly reactive ^1^O_2_ produced in PDT process could in turn deplete intracellular glutathione and activate ferroptosis, and the addition of a redox-responsive nanocarrier could further manipulate the extent of progression (Liu et al., 2018; Meng et al., 2019). Overall, we believe that with the deepening of ferroptosis, photosens, and nanomaterials, the combination therapy of ferroptosis and PDT may provide more new insights into cancer therapy, and even new drugs will be discovered in the future.

### Strengths and Limitations

This is the first-ever study to provide systematic information on ferroptosis-related research through bibliometric analysis coupled with visualized mapping. In order to help new academic researchers to learn about the evolution, current status, and hotspots of ferroptosis research with relative ease, three visualization tools were used to ensure comprehensive assessment. Furthermore, as all the publications were evaluated based on authors, institutes, and countries, our results of this analysis can serve as a reference for scientists and funding agencies to explore potential collaborative partnerships and provide investment guidance.

Several limitations of our study should be pointed out. First, data on ferroptosis publications were only retrieved and collected from WoSCC database, and publications in other databases, such as PubMed, Google Scholar, and Scopus, may not have been identified. Second, it may have overlooked some critical studies published in other languages as only English articles were included in this analysis. Third, although all searches were retrieved on November 12, 2020, to avoid bias due to daily update of WoSCC database, the database remains in the open state as it is continuously receiving new studies. Despite this, we believe that this study has incorporated the vast majority of publications from 2012, and the conclusions would not be changed even with the emerging of a small amount of new data.

## Conclusion

In summary, the study of ferroptosis-related research remains in its early stages. There will be a dramatically increasing number of publications on ferroptosis research based on the current global trends. China has made significant progress in ferroptosis research, but the United States is actually dominated in this field. All the current publications can be divided into two clusters, “pathway and mechanism” and “treatment and effect” of ferroptosis. More focus will be placed on neurodegeneration, chemotherapy, NF-κB, and PDT, which may be the next popular topics in ferroptosis research.

## Author Contributions

HW, HY, and ZS designed the study. LT and YW collected the data. HW, YW, LT, HY, and ZS analyzed the data and drafted the manuscript. HY and ZS revised and approved the final version of the manuscript. All authors contributed to the article and approved the submitted version.

## Conflict of Interest

The authors declare that the research was conducted in the absence of any commercial or financial relationships that could be construed as a potential conflict of interest.

## Footnotes

1
http://bibliometric.com

2
http://www.vosviewer.com/

3
http://cluster.ischool.drexel.edu/~cchen/citespace/download
