# Corrigendum: Global research trends of ferroptosis: a rapidly evolving field with enormous potential

**DOI:** 10.3389/fcell.2023.1270383

**Published:** 2023-08-31

**Authors:** Haiyang Wu, Yulin Wang, Linjian Tong, Hua Yan, Zhiming Sun

**Affiliations:** ^1^ Clinical College of Neurology, Neurosurgery and Neurorehabilitation, Tianjin Medical University, Tianjin, China; ^2^ Tianjin Key Laboratory of Cerebral Vascular and Neurodegenerative Diseases, Tianjin Neurosurgical Institute, Tianjin Huanhu Hospital, Tianjin, China; ^3^ Department of Orthopaedic Surgery, Tianjin Huanhu Hospital, Tianjin, China

**Keywords:** ferroptosis, bibliometric analysis, WoSCC, VOS viewer, CiteSpace

In the published article, there was an error in the legend for Figure 4A as published. One important word is missing. The corrected legend appears below.

**FIGURE 4:**
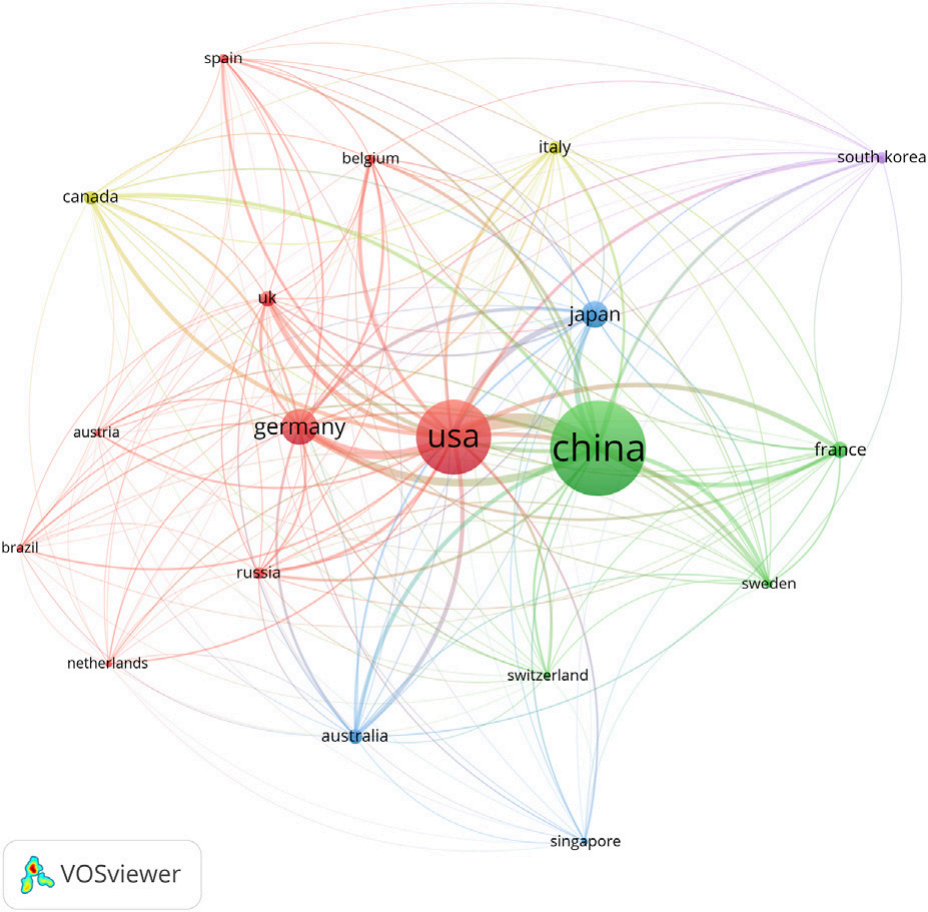
**(A)** The cross-country/region collaborations visualization map. **(B)** The countries/regions citation network visualization map generated by VOS viewer software.

“The cross-country/region collaborations visualization map.”

In the published article, there was an error in Figure 4B as published. An important step was missed when making this graph, thus the published figure was wrong. The corrected Figure 4B and its caption “The countries/regions citation network visualization map generated by VOS viewer software” appear below.

In the published article, there was an error in the **Results** section, *Contributions of Countries/Regions and Funding Agencies*. This sentence previously stated:

“Of the 20 countries/regions that met this threshold, the top three with the largest TLS were the United States (TLS = 18,742), China (TLS = 16,494), and Germany (TLS = 8,289).”

The corrected sentence appears below:

“Of the 19 countries/regions that met this threshold, the top three with the largest TLS were the United States, China, and Germany.”

The authors apologize for this error and state that this does not change the scientific conclusions of the article in any way. The original article has been updated.

