# Supplementary material for: Global Research Trends of Ferroptosis: A Rapidly Evolving Field With Enormous Potential
Source: Front Cell Dev Biol. 2021 Apr 29;9:646311. doi: 10.3389/fcell.2021.646311 (PMC8116802; doi:10.3389/fcell.2021.646311)
Supplement: Supplementary file 1 [file Data_Sheet_1.docx]

Supplementary Material

# Supplementary Figures and Tables

## Supplementary Figure


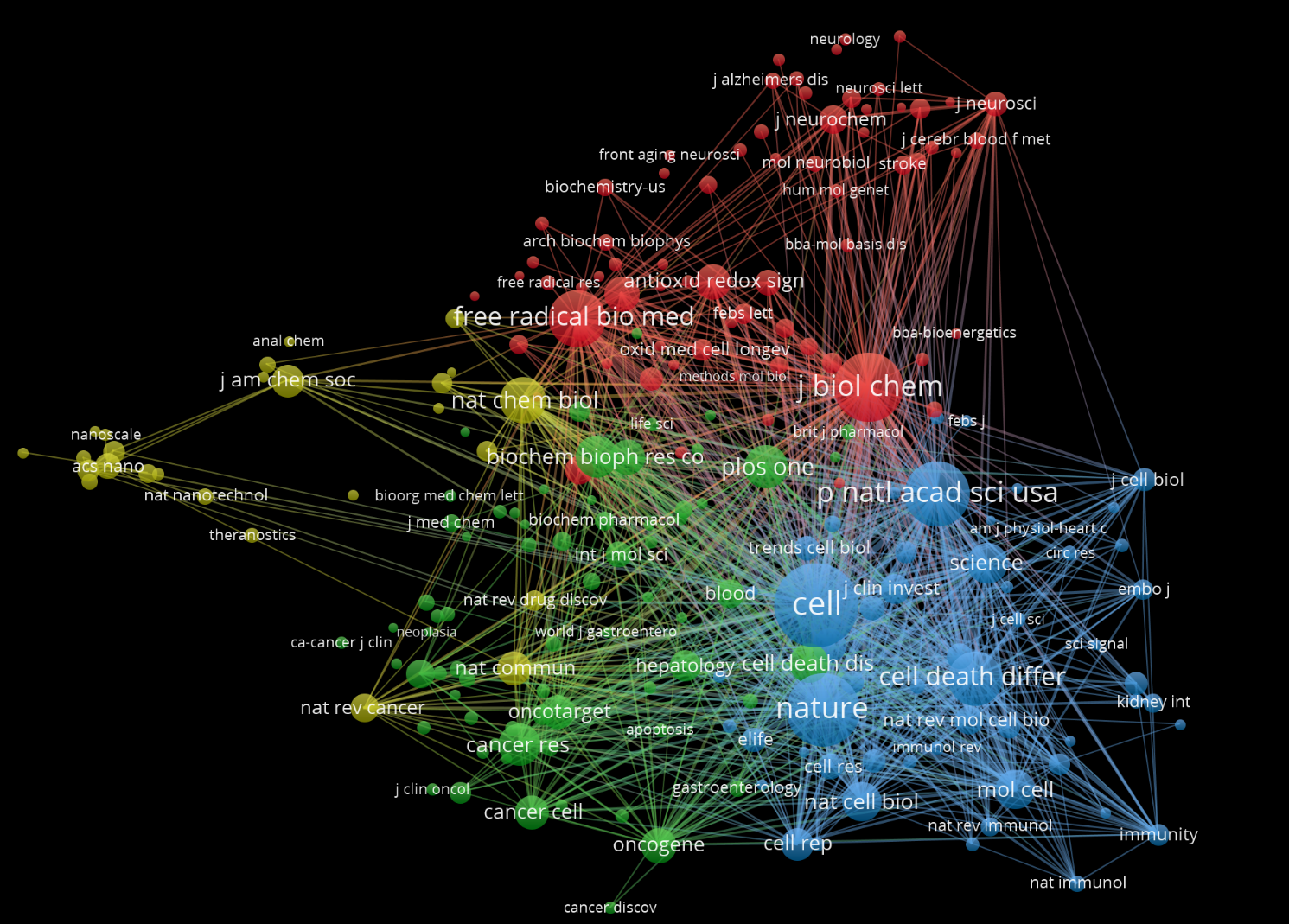


**Supplementary Figure 1.** The journal co-citation network visualization map generated by VOS viewer software.


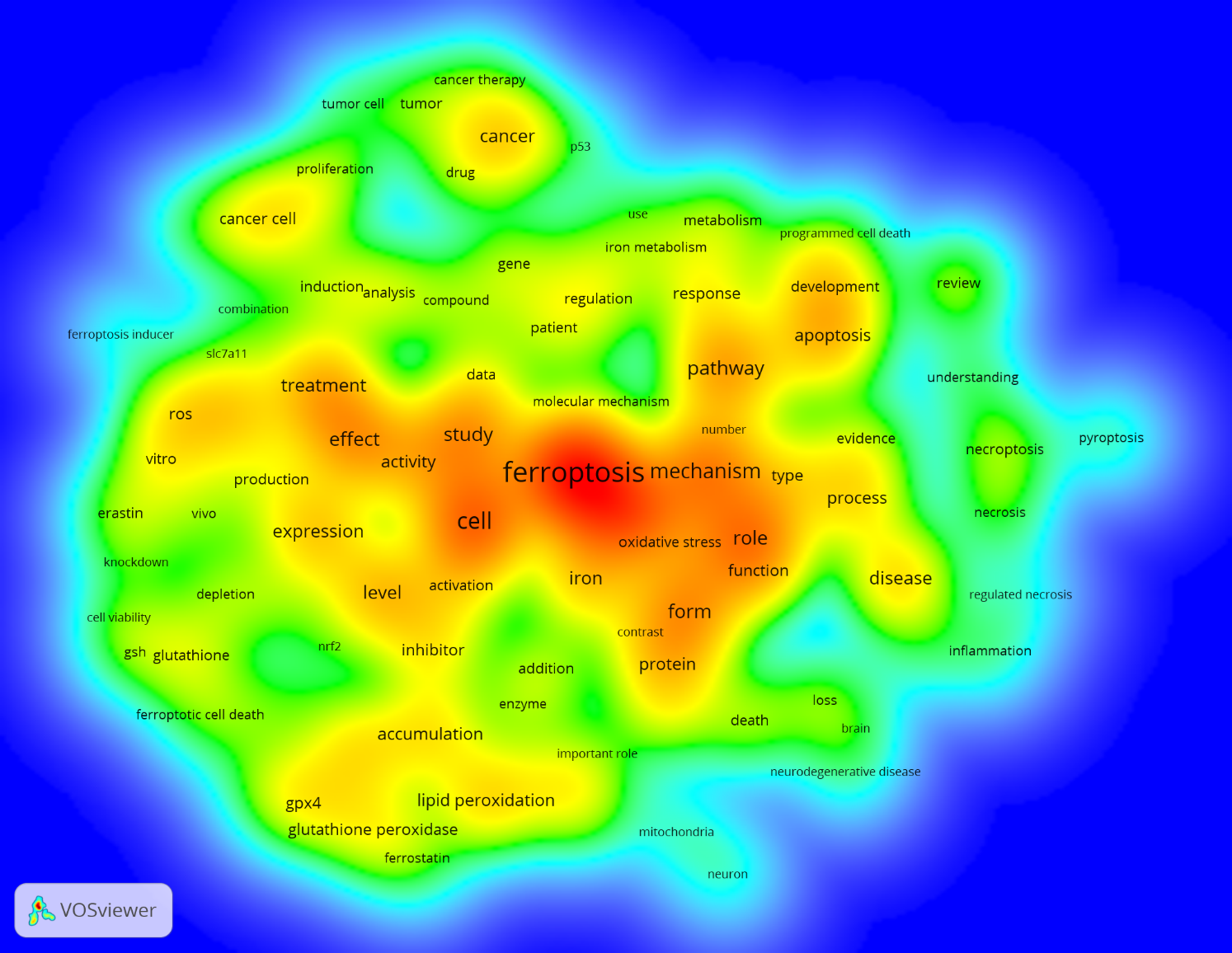


**Supplementary Figure 2.** The density map of keywords generated by VOS viewer software. Keywords with a minimum of 50 occurrences were visualized. A total of 99 Keywords met this threshold.

## Supplementary Tables

**Supplemental Table 1. The top 10 related funding agencies**

| Funding agencies | Countries | Count | Percentage (N/1285) |
| --- | --- | --- | --- |
| National Natural Science Foundation of China (NSFC) | China | 402 | 31.28% |
| National Institutes of Health (NIH) | USA | 261 | 20.31% |
| United States Department of Health Human Services | USA | 261 | 20.31% |
| German Research Foundation (DFG) | German | 72 | 5.60% |
| NIH National Cancer Institute (NCI) | USA | 72 | 5.60% |
| Ministry of Education Culture Sports Science and Technology (MEXT) | Japan | 63 | 4.90% |
| Japan Society for the Promotion of Science | Japan | 49 | 3.81% |
| American Cancer Society | USA | 34 | 2.65% |
| China Postdoctoral Science Foundation | China | 33 | 2.57% |
| National Natural Science Foundation of Guangdong Province | China | 33 | 2.57% |

**Supplemental Table 2. Top 10 ferroptosis related reviews with the most citations (up to March 9, 2021)**

| Title | First Author | Journal | Year | Citations |
| --- | --- | --- | --- | --- |
| Molecular mechanisms of cell death: recommendations of the Nomenclature Committee on Cell Death 2018 | Galluzzi L | *Cell Death Differ* | 2018 | 1150 |
| Ferroptosis: A Regulated Cell Death Nexus Linking Metabolism, Redox Biology, and Disease | Stockwell BR | *Cell* | 2017 | 877 |
| Regulated necrosis: the expanding network of non-apoptotic cell death pathways | Vanden Berghe T | *Nat Rev Mol Cell Biol* | 2014 | 827 |
| The role of iron and reactive oxygen species in cell death | Dixon SJ | *Nat Chem Biol* | 2014 | 745 |
| Ferroptosis: process and function | Xie Y | *Cell Death Differ* | 2016 | 580 |
| Ferrootosis: Death by Lipid Peroxidation | Yang WS | *Trends Cell Biol* | 2016 | 508 |
| Reactive oxygen species and cancer paradox: To promote or to suppress? | Galadari S | *Free Radic Biol Med* | 2017 | 318 |
| Mechanisms of ferroptosis | Cao JY | *Cell Mol Life Sci* | 2016 | 277 |
| Regulated necrosis: disease relevance and therapeutic opportunities | Conrad M | *Nat Rev Drug Discov* | 2016 | 233 |
| The molecular machinery of regulated cell death | Tang D | *Cell Res* | 2019 | 217 |

**Supplemental Table 3. The top 99 keywords in terms of frequency**

| Rank | Keyword | Occurrence | Rank | Keyword | Occurrence | Rank | Keyword | Occurrence |
| --- | --- | --- | --- | --- | --- | --- | --- | --- |
| 1 | ferroptosis | 1053 | 34 | development | 198 | 67 | proliferation | 109 |
| 2 | cell | 724 | 35 | review | 195 | 68 | enzyme | 105 |
| 3 | cell death | 705 | 36 | analysis | 188 | 69 | understanding | 105 |
| 4 | mechanism | 554 | 37 | mouse | 178 | 70 | increase | 104 |
| 5 | role | 499 | 38 | metabolism | 175 | 71 | cancer therapy | 103 |
| 6 | study | 490 | 39 | death | 174 | 72 | ferroptotic cell death | 99 |
| 7 | pathway | 481 | 40 | vitro | 173 | 73 | sensitivity | 97 |
| 8 | form | 444 | 41 | glutathione | 170 | 74 | iron metabolism | 87 |
| 9 | effect | 442 | 42 | necroptosis | 169 | 75 | inflammation | 86 |
| 10 | treatment | 417 | 43 | regulation | 169 | 76 | ability | 85 |
| 11 | level | 390 | 44 | gene | 167 | 77 | pyroptosis | 85 |
| 12 | expression | 358 | 45 | regulated cell death | 164 | 78 | ferroptosis inhibitor | 84 |
| 13 | disease | 347 | 46 | patient | 161 | 79 | tumor cell | 82 |
| 14 | iron | 344 | 47 | induction | 160 | 80 | cell viability | 79 |
| 15 | cancer | 341 | 48 | resistance | 160 | 81 | programmed cell death | 79 |
| 16 | lipid peroxidation | 336 | 49 | addition | 153 | 82 | lipid peroxide | 78 |
| 17 | apoptosis | 328 | 50 | data | 153 | 83 | pathogenesis | 78 |
| 18 | process | 291 | 51 | autophagy | 150 | 84 | combination | 77 |
| 19 | accumulation | 280 | 52 | erastin | 149 | 85 | nrf2 | 77 |
| 20 | protein | 279 | 53 | evidence | 149 | 86 | present study | 77 |
| 21 | inhibition | 276 | 54 | production | 148 | 87 | knockdown | 76 |
| 22 | reactive oxygen species | 274 | 55 | tumor | 141 | 88 | neuron | 74 |
| 23 | activity | 270 | 56 | depletion | 134 | 89 | p53 | 74 |
| 24 | cancer cell | 260 | 57 | drug | 132 | 90 | contrast | 72 |
| 25 | gpx4 | 244 | 58 | loss | 131 | 91 | use | 72 |
| 26 | inhibitor | 242 | 59 | compound | 128 | 92 | neurodegenerative disease | 70 |
| 27 | function | 235 | 60 | change | 122 | 93 | number | 70 |
| 28 | ros | 233 | 61 | ferrostatin | 122 | 94 | slc7a11 | 70 |
| 29 | type | 231 | 62 | necrosis | 121 | 95 | mitochondria | 68 |
| 30 | response | 223 | 63 | gsh | 119 | 96 | ferroptosis inducer | 66 |
| 31 | glutathione peroxidase | 222 | 64 | progression | 118 | 97 | important role | 61 |
| 32 | activation | 199 | 65 | vivo | 117 | 98 | brain | 60 |
| 33 | oxidative stress | 199 | 66 | molecular mechanism | 109 | 99 | regulated necrosis | 57 |

Keywords with a minimum of 50 occurrences were included. A total of 99 Keywords met this threshold.

**Supplemental Table 4. Summary of ferroptosis involved in multiple cancers**

| Cancer types | Related pathway/Target | Main conclusion | References |
| --- | --- | --- | --- |
| Hepatocellular carcinoma | NRF2 | The inhibition of NRF2 expression/activity in hepatocellular carcinoma cells could increase the anticancer activity of erastin and sorafenib in vitro and in tumor xenograft models. | Sun et al. (2016). *Hepatology*. 63(1), 173-184. |
| Lung cancer | NFS1 | Suppression of NFS1 cooperated with inhibition of cysteine transport to trigger ferroptosis in vitro and slow tumour growth. | Alvarez et al. (2017). *Nature.*551(7682), 639-643. |
|  | FSP1 | FSP1 mediated resistance to ferroptosis in lung cancer cells and in mouse tumor xenografts | Bersuker et al. (2019). *Nature.* 575(7784), 688-692 |
| Ovarian cancer | Iron metabolism | The iron dependence of ovarian cancer tumor-initiating cells made them exquisitely sensitive in vivo to ferroptosis inducers and iron chelators | Basuli et al. (2017). *Oncogene*. 36(29), 4089-4099. |
|  | TAZ | TAZ promoted ferroptosis in ovarian cancers by regulating ANGPTL4 and NOX. | Yang et al. (2020). *Mol Cancer Res*. 18(1), 79-90. |
| Cervical cancer | HSPB1 | Knockdown of HSPB1 and HSF1 could enhance erastin-induced ferroptosis. | Sun et al. (2015). *Oncogene*. 34(45), 5617-5625. |
| Breast cancer | FPN | Overexpression FPN resulted in decreased ROS and ferroptosis whereas knockdown of FPN increased ferroptosis after breast cancer treatment with siramesine and lapatinib. | Ma et al. (2016). *Cell Death Dis*. 7(7), e2307. |
| Urologic neoplasms | GSH/GPX | Renal cancer cells highly dependent on the GSH/GPX pathway to prevent lipid peroxidation and ferroptotic cell death. | Miess et al. (2018). *Oncogene*. 37(40), 5435-5450. |
|  | TAZ | Cell density-regulated ferroptosis in renal cell carcinoma was mediated by TAZ through the regulation of EMP1-NOX4. | Yang et al. (2019). *Cell Rep*. 28(10), 2501-2508.e4. |
|  | ZNF217 | Elevated expression of ZNF217 promoted the growth of prostate cancer by inhibiting FPN-conducted iron egress. | Jiang et al. (2016). *Oncotarget*. 7(51), 84893-84906. |
| Pancreatic cancer | System Xc^-^ | Pancreatic ductal adenocarcinoma cells used cysteine to synthesize glutathione and coenzyme A to down-regulated ferroptosis. | Badgley et al. (2020). *Science*. 368(6486), 85-89. |
|  | HSPA5 | HSPA5 negatively regulated ferroptosis in human pancreatic ductal adenocarcinoma. | Zhu et al. (2017). *Cancer Res*. 77(8), 2064-2077. |
| Glioma | Nrf2-Keap1 | Both fostered Nrf2 expression and conversely Keap1 inhibition promoted resistance to ferroptosis in gliomas. | Fan Z et al. (2017). *Oncogenesis*. 2017;6(8), e371. |
| Glioblastoma | System Xc^-^ | When co-treatment with erastin, Temozolomide‑induced cytotoxicity was significantly increased with a marked decrease of GSH levels. | Chen et al. (2015). *Oncol Rep*. 33(3), 1465-1474. |
| Haematological neoplasms | LOX | Lipoxygenases contributed to the regulation of RSL3-induced ferroptosis in acute lymphoblastic leukemia cells. | Probst et al. (2017). *Biochem Pharmacol*. 140, 41-52. |
|  | AMPK/mTOR/p70S6k | DHA promoted the accumulation of cellular ROS and eventually led to ferroptotic cell death by regulating the activity of AMPK/mTOR/p70S6k signaling pathway in acute myeloid leukemia. | Du et al. (2019). *Free Radic Biol Med*. 131, 356-369. |
|  | GPX4 | Diffuse large B cell lymphomas were particularly susceptible to GPX4-regulated ferroptosis | Yang et al. (2014). *Cell*. 156(1-2), 317-331. |
| Colorectal cancer | DPP4 | TP53 limited erastin-induced ferroptosis by blocking DPP4 activity in a transcription-independent manner | Xie et al. (2017). *Cell Rep*. 20(7), 1692-1704. |
| Head and neck cancer | System Xc^-^ | Inhibition of cystine/glutamate antiporter overcame the cisplatin resistance of head and neck cancer cells by inducing ferroptosis. | Roh et al. (2016). *Cancer Lett*. 381(1), 96-103. |
| Osteosarcoma | STAT3/Nrf2/GPx4 | Ferroptosis induced by impairing STAT3/Nrf2/Gpx4 signal pathway enhanced the sensitivity of osteosarcoma cells to cisplatin | Liu et al. (2019). *Cell Biol Int*. 43(11), 1245-1256. |
